# Measurement Invariance of the WISC-V across a Clinical Sample of Children and Adolescents with ADHD and a Matched Control Group

**DOI:** 10.3390/jintelligence12010006

**Published:** 2024-01-12

**Authors:** Angelika Beate Christiane Becker, Jenny Maurer, Monika Daseking, Franz Pauls

**Affiliations:** 1 Department of Developmental and Educational Psychology, Helmut Schmidt University, 22043 Hamburg, Germany; maurerj@hsu-hh.de (J.M.); dasekinm@hsu-hh.de (M.D.); 2 Department of Clinical Psychology and Psychotherapy, Helmut Schmidt University, 22043 Hamburg, Germany; paulsf@hsu-hh.de

**Keywords:** Wechsler Intelligence Scale for Children, fifth edition, measurement invariance, ADHD, clinical sample

## Abstract

Measurement invariance of the Wechsler Intelligence Scale for Children, Fifth Edition (WISC-V) 10-primary subtest battery was analyzed across a group of children and adolescents with ADHD (*n* = 91) and a control group (*n* = 91) matched by sex, age, migration background, and parental education or type of school. First, confirmatory factor analyses (CFAs) were performed to establish the model fit for the WISC-V second-order five-factor model in each group. A sufficiently good fit of the model was found for the data in both groups. Subsequently, multigroup confirmatory factor analyses (MGCFAs) were conducted to test for measurement invariance across the ADHD and control group. Results of these analyses indicated configural and metric invariance but did not support full scalar invariance. However, after relaxing equality constraints on the Vocabulary (VC), Digit Span (DS), Coding (CD), Symbol Search (SS), and Picture Span (PS) subtest intercepts as well as on the intercepts of the first-order factors Working Memory (WM) and Processing Speed (PS), partial scalar invariance could be obtained. Furthermore, model-based reliability coefficients indicated that the WISC-V provides a more precise measurement of general intelligence (e.g., represented by the Full-Scale IQ, FSIQ) than it does for cognitive subdomains (e.g., represented by the WISC-V indexes). Group comparisons revealed that the ADHD group scored significantly lower than the control group on four primary subtests, thus achieving significantly lower scores on the corresponding primary indexes and the FSIQ. Given that measurement invariance across the ADHD and the control group could not be fully confirmed for the German WISC-V, clinical interpretations based on the WISC-V primary indexes are limited and should only be made with great caution regarding the cognitive profiles of children and adolescents with ADHD.

## 1. Introduction

Attention deficit hyperactivity disorder (ADHD) is one of the most common neurodevelopmental disorders in childhood and adolescence, with an estimated prevalence rate of approximately 5% (prevalence rates between 2% and 7% are commonly reported) (Huss et al. 2008; Mohammadi et al. 2021; Polanczyk et al. 2007; Sayal et al. 2018). ADHD manifests as a consistent behavioral pattern of inattention, hyperactivity, and/or impulsivity that occurs across various environments, such as at home and school, and can lead to severe difficulties in social, educational, or occupational settings (Harpin 2005). In the *Diagnostic and Statistical Manual of Mental Disorders* (5th ed.; DSM-5; American Psychiatric Association 2013) three major types of ADHD are specified according to symptomatology: ADHD with predominantly inattentive symptoms, with predominantly hyperactive-impulsive symptoms, or with combined symptoms. ADHD is also associated with specific cognitive deficits or with a specific profile of cognitive performance described in more detail in the following paragraph.

### 1.1. Cognitive Profiles of Children and Adolescents with ADHD

Theories pertaining to ADHD propose that this mental disorder is accompanied by fundamental deficits in behavioral inhibition that involves the capacity to restrain pre-existing or interfering responses, subsequently affecting other cognitive domains (Barkley 1997). In children and adolescents with ADHD, cognitive deficits have mainly been found regarding working memory (Kasper et al. 2012; Martinussen et al. 2005) and processing speed (Gau and Huang 2014; Salum et al. 2014; Shanahan et al. 2006). However, findings of an extensive meta-analysis suggest a set of additional deficits across a variety of neurocognitive domains to be associated with ADHD, including reaction time variability, response inhibition, intelligence/achievement, and planning/organization (Pievsky and McGrath 2018). Further meta-analytical studies point toward lower levels of cognitive abilities in children and adolescents with ADHD (Frazier et al. 2004; Pievsky and McGrath 2018). However, achieving lower scores on intelligence tests (IQ tests) might not only be attributed to decreased working memory capacity or processing speed in individuals with ADHD, but may also be caused by additional attention deficits and increased levels of impulsivity during the test administration itself (Jepsen et al. 2009).

### 1.2. Obtaining Cognitive Measures and Profiles for Children and Adolescents with ADHD

According to the clinical guidelines for diagnosing ADHD by the American Academy of Pediatrics, conducting neuropsychological tests when diagnosing ADHD may generally provide an in-depth evaluation of a child’s or adolescent’s learning strengths and weaknesses (Wolraich et al. 2019). In particular, testing for cognitive abilities is recommended as part of this diagnostic process in order to rule out cognitive over- or underachievement, which could also cause symptoms similar to those associated with ADHD (Döpfner et al. 2013; Drechsler et al. 2020; Pliszka 2007). Moreover, the need to estimate and discriminate between different levels of cognitive abilities in this context is additionally underlined by the high comorbidity between symptoms of ADHD and learning disorders (Mattison and Mayes 2012; Mayes et al. 2000; Sexton et al. 2012). The Wechsler scales are among the most common test batteries used worldwide by clinicians to assess specific domains of cognitive abilities in children and adolescents with ADHD (Becker et al. 2021; Mayes and Calhoun 2006; Scheirs and Timmers 2009). Various studies using different versions of the Wechsler Intelligence Scale for Children (WISC), such as the WISC-III (Wechsler 1991), WISC-IV (Wechsler 2003) or WISC-V (Wechsler 2014b), have already indicated significant deficits in individuals with ADHD by reporting lower scores on the working memory and processing speed indexes (Assesmany et al. 2001; Becker et al. 2021; Devena and Watkins 2012; Mayes and Calhoun 2006; Mealer et al. 1996; Yang et al. 2013). Since a study with a German speaking clinical sample of children and adolescents with ADHD indicates that such specific deficits can be appropriately identified using the WISC-V (Pauls et al. 2018), a comprehensive intelligence test battery might be a valid instrument when assessing strengths and weaknesses in the cognitive profiles of children and adolescents with ADHD.

### 1.3. Structural Framework of the WISC-V

The conceptual and structural framework of the WISC-V (Wechsler 2014b) is based on the Cattell–Horn–Carrol (CHC) model of intelligence (McGrew 2009; Schneider and McGrew 2012), thus providing an encompassing taxonomy of neuropsychological constructs (Wechsler 2014a). As a major change to the previous test version, the WISC-V redefines the four-factor structure underlying the WISC-IV into a new hierarchical five-factor model structure. As suggested by the factor analytical findings provided in the WISC-V technical manuals of both the German and US versions of the WISC-V (Wechsler 2014a, 2017a), this five-factor model structure (referred to as second-order five factor model) is proposed to be an adequate representation of the underlying nature of intelligence as described by the CHC model. In addition to the Visual Spatial Index (VSI) and the Fluid Reasoning Index (FSI), which replaced the former WISC-IV Perceptual Reasoning Index (PRI), the Verbal Comprehension Index (VCI), the Working Memory Index (WMI), and the Processing Speed Index (PSI) can also be determined in the WISC-V. Scaled scores for each of those five primary indexes are derived by utilizing two out of a total of ten primary subtests: Similarities (SI), Vocabulary (VC), Block Design (BD), Matrix Reasoning (MR), Figure Weights (FW), Visual Puzzles (VP), Digit Span (DS), Picture Span (PS), Coding (CD), and Symbol Search (SS). The Full-Scale Intelligence Quotient (FSIQ) is derived on the basis of seven primary subtests, while three additional primary subtests are required to calculate all five primary indexes. The FSIQ is defined as an estimate of the overall cognitive ability and represents a global measure for the five underlying cognitive subdomains, which, on their part, are represented by the WISC-V primary indexes.

### 1.4. Measurement Invariance of the WISC-V across Different Groups

In order to be able to compare test scores of individuals from different populations or groups in a meaningful and reliable way, it is essential for any diagnostic instrument used to provide measures that have the same meaning across those groups in question (e.g., Chen and Zhu 2012; Millsap and Kwok 2004; Wicherts 2016). Given that the standardization of diagnostic instruments is often based on population-representative data, construct validity of such instruments can be significantly affected when conducted on different populations or specific clinical groups (Chen and Zhu 2012). Thus, providing measurement invariance is a crucial requirement to ensure test fairness. It is only when measurement invariance can be established that individual differences in test scores may be adequately interpreted as true variations in the underlying cognitive domains (Chen et al. 2005). Multigroup confirmatory factor analysis (MGCFA) is the most commonly used technique for investigating measurement invariance across different groups. In MGCFA, a theoretical model is compared with the observed structures in multiple independent samples (Vandenberg and Lance 2000). Testing for measurement invariance requires a stepwise approach, in which nested models are sequentially analyzed with additional constraints being imposed on each subsequent model (Jöreskog 1993; Pauls et al. 2013).

MGCFA has already been used on normative sample data to test for measurement invariance across sex and different age groups (Chen et al. 2020; Pauls et al. 2020; Reynolds and Keith 2017; Scheiber 2016). However, studies investigating measurement invariance of the WISC-V across clinical groups, such as children and adolescents with ADHD, are sparse and more research on such groups is needed (Chen et al. 2020). Bowden et al. (2008) tested measurement invariance of the Wechsler Adult Intelligence Scale–III (WAIS-III; Wechsler 1997) in three different groups: a group of college students with learning disabilities, a group of college students with ADHD, and an age-matched cohort with no diagnosis. While measurement equivalence could be demonstrated, there were significant differences between the groups with respect to the variances, covariances, and means of the underlying latent factors. Another clinical study investigated the factor structure and tested for measurement invariance of the ten WISC-V primary subtests across a group of children and adolescents with a diagnosis of autism spectrum disorder (ASD) and a healthy control group (Stephenson et al. 2021). Here, measurement invariance could only be partially established, as the WISC-V primary subtests Coding and Digit Span were found to be not invariant across the ASD and the healthy control group. Dombrowski et al. (2021) analyzed measurement invariance of the WISC-V across sex, four different age groups, and three different clinical groups (ADHD, anxiety disorders, and encephalopathy). Although they could demonstrate full invariance across sex and the clinical groups, only partial invariance was found across the age groups under examination. However, Dombrowski et al. (2021) compared three different clinical groups (ADHD, anxiety, and encephalopathy), but did not provide a comparison with a matched healthy control group. In this regard, the current study may contribute towards closing a research gap. Due to a lack of comparable studies specifically focusing on the structural validity of the WISC-V in individuals with ADHD, the aim of the present study was to investigate measurement invariance of the second-order five-factor model of the German WISC-V (Wechsler 2017b) across a sample of children and adolescents with ADHD and a matched healthy control group. Given that model-based reliability and group differences were additionally analyzed, this overall analytical approach should help to clarify whether the WISC-V model suggested by the test publishers is fully or partially transferable to an ADHD population.

## 2. Materials and Methods

### 2.1. Sample Characteristics

Children and adolescents who had received a confirmed diagnosis of ADHD or attention deficit disorder (ADD) were selected by a cooperating child and adolescent psychiatric outpatient clinic using disorder-specific diagnostic instruments and were screened for further eligibility criteria. For the latter, exclusion criteria included a general IQ score of less than 70, severe neurological or psychological impairments, severe auditory, visual, or motor impairments, and insufficient German language skills to follow the instructions of the WISC-V (Wechsler 2017b). Data of *n* = 91 children and adolescents, respectively, *n* = 26 females (28.6%) and *n* = 65 males (71.4%), with a mean age of 10.83 years (*SD* = 2.47; age range = 7.0–16.6 years), were gathered for conducting single-group and multigroup confirmatory factor analyses. In total, *n* = 14 (15.4%) children and adolescents had a diagnosis of ADD and *n* = 77 (84.6%) met the diagnostic criteria of ADHD. *N* = 63 children and adolescents had a comorbid diagnosis of a learning disorder (69.2%). For the healthy control group of the present study, data were selected from the German WISC-V standardization sample of children and adolescents with no reported indication of a diagnosed ADHD/ADD or learning disorder to match the ADHD group for sex, age, migration background, and parental educational level for the younger children (aged 6–9 years) or type of school for the older children and adolescents (aged 10–16 years). Demographic characteristics of the ADHD and control group are depicted in Table 1.

The ADHD group and the control group were compared in terms of demographic variables. A t-test indicated that both groups did not differ in respect to their ages, *t*(180) = −.959, *p* = .342. Chi-square tests were calculated to test whether the distribution of sex or migration status differed between the two groups, which was neither the case with sex, χ²(1) = 0.00, *p* = 1.000, φ = 0.00, nor with migration status, χ²(1) = 0.27, *p* = .870, φ = 0.12. The distributions of the variables parental educational background and type of school were compared between the groups using the Mann–Whitney U-test. There was no statistically significant group difference in parental educational background, *U* = 4009.00, *Z* = −.255, *p* = .798, nor in type of school, *U* = 3739.00, *Z* = −1.096, *p* = .273.

### 2.2. Measurement Instruments

Following the guidelines of a standardization kit that provides the basic framework for all European WISC-V versions, the German WISC-V (Wechsler 2017b) was adapted from the original US version (WISC-V USA; Wechsler 2014b) to provide a comprehensive test of intelligence. Unlike the original version including 21 subtests, the German WISC-V comprises a total of 15 subtests, including the ten primary subtests Similarities (SI), Vocabulary (VC), Block Design (BD), Matrix Reasoning (MR), Figure Weights (FW), Visual Puzzles (VP), Digit Span (DS), Picture Span (PS), Coding (CD), and Symbol Search (SS), from which seven primary subtests (BD, SI, MR, DS, CD, VC, and FW) are used to derive the Full-Scale Intelligence Quotient (FSIQ). The scaled scores of all ten primary subtests (*M* = 10, *SD* = 3) are required to calculate the five primary index scores for Verbal Comprehension (VCI), Visual Spatial (VSI), Fluid Reasoning (FRI), Working Memory (WMI), and Processing Speed (PSI). These primary index scores, as well as the FSIQ, are defined by standard scores on the IQ scale (*M* = 100, *SD* = 15). Although an excellent internal consistency with Cronbach’s alpha values ranging from 0.81 to 0.93 has already been demonstrated for the primary subtests of the German WISC-V (Wechsler 2017a), omega-hierarchical and omega-hierarchical subscale coefficients have been frequently recommended as more appropriate reliability measures for hierarchical model structures (Brunner et al. 2012; Reise 2012; Sijtsma 2009; Yang and Green 2011). Therefore, the according model-based reliability coefficients are additionally described in detail in the following subsection and reported in the results section of the present article.

### 2.3. Analytical Procedures

#### 2.3.1. Single-Group Confirmatory Factor Analyses (Phase 1)

Since confirmatory factor analyses have already indicated a hierarchical model solution to satisfactorily represent the factorial structure of the German WISC-V (for EFA and CFA analyses and for a visualization of the model on the 10 primary subtests (see Pauls and Daseking 2021; Wechsler 2017a), the second-order five-factor model (e.g., with five first-order factors representing the five primary indexes) proposed by the test publishers was used as a baseline model for all subsequent analyses. Initially and prior to measurement invariance analyses, the second-order five-factor model was tested for the ADHD group and the matched control group separately in order to test its overall fit in both groups (Phase 1). The formal scoring procedure reported in the *WISC-V Technical and Interpretive Manual* (Wechsler 2014a, p. 83) was applied to specify the baseline model. The latter includes scaled scores of the ten WISC-V primary subtests as indicator variables for five first-order factors and one second-order factor. Reflecting specific cognitive abilities, the first-order factors are then suggested to be best represented by the five WISC-V primary indexes: VCI indicated by scaled scores on the subtests SI and VC, VSI derived using scaled scores on the subtests BD and VP, FRI indicated by scaled scores on the subtests MR and FW, WMI composed of scaled scores on the subtests DS and PS, and PSI derived using scaled scores on the subtests CD and SS. The second-order factor was specified to account for the intercorrelations among the five first-order factors and was thus best represented by the FSIQ.

#### 2.3.2. Multigroup Confirmatory Factor Analyses (Phase 2)

Analyses of measurement invariance across the ADHD and matched control group were based on the variance–covariance structure of the underlying data and were conducted using AMOS 29 (Arbuckle 2022). For all confirmatory factor analyses required, scales of latent variables were identified by fixing one factor loading of each latent variable to one (Keith 2015). Since scaled scores were used for all measurement invariance analyses, each subtest was initially checked for normality. In the ADHD group, skewness for the scaled scores on the ten WISC-V primary subtests ranged from −0.50 to 0.34 and kurtosis ranged from −0.65 to 0.47, with a multivariate kurtosis of −1.28. In the matched control group, skewness for the scaled scores ranged from −0.79 to 0.35 and kurtosis ranged from −0.51 to 0.93, with a multivariate kurtosis of 2.62. Since skewness and kurtosis values did not indicate any excessive deviation from normality (see West et al. 1995, for an overview), maximum likelihood was used as a robust procedure for model estimation.

Provided that a reasonable fit of the hypothesized second-order five-factor model could be established for the ADHD and matched control group individually by conducting single-group confirmatory factor analyses (Phase 1), measurement invariance across both groups could then be evaluated by testing different invariance levels using multigroup confirmatory factor analyses in Phase 2 (Keith 2015). Following a hierarchical structure for testing measurement invariance, each level was specified and analyzed within subsequent models with decreasing numbers of parameters to be estimated due to the inclusion of parameter constraints, one at a time. Given that each subsequent model and its corresponding parameter constraints were nested in the previous model, measurement invariance models then became increasingly more restrictive.

At the first and weakest level of invariance (configural invariance), invariance was evaluated based on whether the overall baseline model (M_1_) appeared to be equally structured across the ADHD and control group (e.g., equal numbers and patterns of factors). Once configural invariance could be established, another model was specified at the second level of invariance to test whether both groups responded to the test items in the same way (metric invariance). This model (M_2_) was specified by constraining all loadings of the subtest indicators on the associated first-order factors to be equal across both groups (first-order metric invariance). In a subsequent model at the third level of invariance (M_3_), all second-order factor loadings were additionally constrained to be equal across both groups in order to test whether the scales of the latent factors as well as the units of measurement could be characterized as invariant across the ADHD and matched control group (second-order metric invariance). For the next model at the fourth level of invariance (M_4_), all subtest intercepts were additionally constrained to be equal across the ADHD and matched control group (scalar invariance for the observed indicator variables). Scalar invariance would indicate that examinees with the same score on a certain latent variable would obtain the same score on the observed variable irrespective of their group membership. To evaluate whether mean scores of the first-order factors might be considered comparable across the ADHD and matched control group, a subsequent model was specified at the fifth level of invariance (M_5_) by additionally constraining the intercepts of all first-order factors to equality across both groups (scalar invariance for the first-order factors). A final model was specified at the sixth level of invariance (M_6_) to examine the equivalence of variances in measurement errors by constraining the error terms of the observed variables to be equal across the ADHD and control group (residual invariance). Establishing residual invariance would then indicate that all group-related differences on the indicator variables were attributable to group-related differences on the corresponding first-order factors.

The evaluation of each measurement invariance model was based on a preselected set of model fit indexes in order to overcome the limitations of each single index (see Kline 2016; McDonald and Ho 2002; Thompson 2000, for an overview). Accordingly, model evaluation was based on the examination of absolute fit indexes such as the likelihood ratio chi-square statistic (χ^2^), the standardized root mean square residual (SRMR), and the root mean square error of approximation (RMSEA). The overall model fit was considered acceptable if χ^2^ was found not to be significant. According to Hu and Bentler (1999), an SRMR value of zero indicates a perfect fit, whereas values less than .05 correspond to a good fit, and a value of .08 indicates an acceptable fit to the data. RMSEA values less than or equal to .01 indicate an excellent fit and a value of .05 corresponds to a good fit, whereas values greater than or equal to .10 indicate a poor model fit (MacCallum et al. 1996). Along with the aforementioned absolute fit indexes, parsimonious fit indexes were also examined for model evaluation, including the chi-square to degrees-of-freedom ratio (χ^2^/df), with a ratio of 5:1 or less corresponding to an acceptable model fit (Schumacker and Lomax 2004), and the comparative fit index (CFI), with values above .95 indicating a good fit (Hu and Bentler 1999). Finally, the Akaike information criterion (AIC) was examined to compare nested and non-nested models, with lower values representing a better model fit (Kaplan 2000).

In line with the criteria commonly used for determining evidence of measurement invariance (Byrne and Stewart 2006), invariance models were partly evaluated by examining differences between χ^2^ values of successive models (Δχ^2^) to test whether the absolute fit of a more restrictive invariance model was significantly lower than for the less restrictive model. A non-significant Δχ^2^ value implies that both invariance models fit the data equally well. Additionally, a change in RMSEA values between successive models (ΔRMSEA) greater than .015 was also determined as an indication of a meaningful drop in model fit (Chen 2007). The change in CFI values (ΔCFI) was also examined in order to provide a measure of invariance that was relatively independent of sample sizes and model complexities (Cheung and Rensvold 2002). As recommended for ΔCFI, values above .01 were regarded as an indication of an unacceptable deterioration in model fit. For the overall evaluation of each single level of invariance to be as unbiased and reasonable as possible, Δχ^2^ and ΔCFI tests were jointly evaluated. However, if changes in both fit indexes indicated contrary results, the overall evaluation was primarily based on the more liberal ΔCFI value (Kline 2016). In cases where full invariance was rejected on a certain level of invariance based on the aforementioned criteria, an examination of partial invariance was consciously considered and subsequent models were based on partial invariance (e.g., Byrne and Watkins 2003). For this purpose, an improvement in the inadequate model fit was intended by relaxing those non-invariant model parameters, which were indicated by the critical ratios for pairwise parameter comparisons provided by AMOS 29 (Arbuckle 2022).

#### 2.3.3. Model Parameter Estimations and Model-Based Reliability

Model-based reliability estimates have often been proposed as an alternative measure of reliability for structural equation modeling (Brunner et al. 2012; Reise 2012; Rodriguez et al. 2016) due to the practical limitations of Cronbach’s alpha (Dunn et al. 2014; Sijtsma 2009; Yang and Green 2011). Thus, omega (ω), omega-hierarchical (ω_H_), and omega-hierarchical subscale (ω_HS_) coefficients have been deemed to provide an appropriate estimation of reliability for multidimensional constructs (e.g., Canivez 2016; McDonald 1999). On the one hand, reliability analyses using ω are based on the proportion of total systematic variance in each factor attributed to the blend of general and subscale variance. On the other hand, ω_H_ indicates the reliability of the higher-order factor adjusted for the subscale variance and ω_HS_ indicates the reliability of each lower-order factor independent of the general factor variance as well as all other subscale variances.

Despite being commonly referred to as reliability estimates, ω coefficients may also enable an evaluation of whether specific factors included in the model can, or even should, be interpreted in a meaningful way (Dombrowski et al. 2018). In the present study, model-based reliability was thus analyzed using ω_H_ and ω_HS_ coefficients in order to determine whether the WISC-V primary index scores can be considered to precisely reflect the underlying cognitive domains and whether additional information above and beyond the FSIQ can be provided by scores at the index level (see Rodriguez et al. 2016 for an application). A robust ω_HS_ coefficient, for instance, might indicate that most of the variance in the primary subtests can be explained by the corresponding WISC-V primary index independent of the FSIQ. Conclusively, individual cognitive abilities may then be interpreted more specifically at the index level (Brunner et al. 2012). By contrast, low values of ω_HS_ would imply that most of the reliable variance is instead explained by the FSIQ. In the latter case, the WISC-V primary indexes would provide rather insufficient representations of specific cognitive domains and interpretations on the index level would likely be flawed (Rodriguez et al. 2016). According to general recommendations, ω_H_ and ω_HS_ values near .750 are preferred, and values should not fall below .500 (Reise et al. 2013).

Along with ω_H_ and ω_HS_, the *H* coefficient was additionally calculated as a measure of construct replicability in order to estimate whether latent variables were adequately represented by the associated indicator variables (Hancock and Mueller 2001). *H* values should not be less than .700 to indicate that indicator variables are useful for stable replications of latent variables across studies (Hancock and Mueller 2001; Rodriguez et al. 2016). Both ω and *H* coefficients as well as other sources of variance were obtained using the Omega program (Watkins 2013) according to an orthogonalized higher-order factor model with five first-order factors. For this purpose, decomposed variance sources from the second-order five-factor model were initially derived using the SL procedure provided by the MacOrtho program (Watkins 2004).

#### 2.3.4. Group Comparisons

Finally, mean and distributional differences in the subtest scaled scores, index scores, and the FSIQ were analyzed across the ADHD and control group by conducting a set of unpaired t-tests. The significance level of the analyses was determined as α = .05. The alpha level for multiple comparisons was adjusted by using the Bonferroni–Holm method. Furthermore, effect sizes for group differences were indicated by Cohen’s d that was interpreted according to Cohen (1988) as follows: *d* = 0.20 indicating a small, *d* = 0.50 indicating a medium, and *d* = 0.80 indicating a large effect size.

## 3. Results

### 3.1. Single-Group Confirmatory Factor Analyses (Phase 1)

The WISC-V subtest variance-covariance matrix was used for model identification, and goodness-of-fit statistics for the WISC-V second-order five-factor model for the ADHD and control group are depicted in Table 2. Although the χ^2^ statistics suggested a slightly insufficient model fit to the observed data in the control group, all other fit indexes indicated a sufficiently good fit of the hypothesized second-order five-factor model to the data of both groups. Since the majority of fit indexes were in an acceptable range, the WISC-V factor structure was deemed to be similar for both groups and was thus selected as the baseline (configural) model for the subsequent measurement invariance analyses.

### 3.2. Multigroup Confirmatory Factor Analyses (Phase 2)

Measurement invariance was examined by conducting a sequence of multigroup confirmatory factor analyses (MGCFA) on nested invariance models in a stepwise manner. Multigroup goodness-of-fit indexes and statistics for each invariance model as well as the model comparisons are summarized in Table 3. First, configural invariance (M_1_) was tested by comparing the unconstrained baseline model across the ADHD and control group simultaneously. Since the baseline model M_1_ provided a good fit to the data (CFI = .971, SRMR = .055, and RMSEA = .038), configural invariance could be accepted, indicating the equal WISC-V factor patterns with subtest loadings on the same corresponding latent factors for both groups.

Given that configural invariance could be established, metric invariance (M_2_) was next tested by constraining all first-order factor loadings to be equal across both groups in M_1_. Since the fit indexes indicated a good fit for M_2_ (CFI = .970, SRMR = .060, and RMSEA = .039) and a model comparison between M_2_ and M_1_ did not suggest any significant deterioration of fit (ΔRMSEA = .001; ΔCFI = −.001; Δχ^2^ = 4.709, Δ*df* = 5, *p* = .452), first-order loadings could be suggested to be comparable across both groups. In order to complement the overall metric invariance examination, additionally constraining all loadings on the second-order factors to be equal across both groups (M_3_) did not result in a significantly worse model fit compared to M_2_ (ΔRMSEA = .001; ΔCFI = −.002; Δχ^2^ = 5.363, Δ*df* = 4, *p* = .252). Thus, metric invariance could be established, suggesting that the strengths of the linear relationships between the second-order factor and the underlying five first-order factors were comparable across both groups.

**Figure 1:**
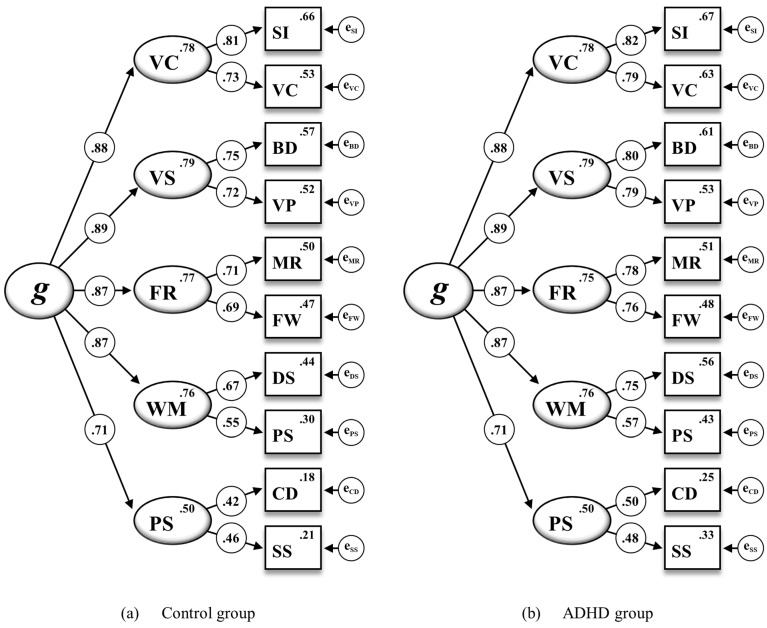
The WISC-V second-order five-factor model for the ADHD and control group including standardized estimations of all regression weights, subtest intercepts, and intercepts of the first-order factors for the 10 WISC-V primary subtests (M_4_ in Table 3). Note. Second-order factor: *g* = general intelligence. First-order factors: VC = Verbal Comprehension, VS = Visual Spatial, FR = Fluid Reasoning, WM = Working Memory, PS = Processing Speed. Subtest indicators: SI = Similarities, VC = Vocabulary, BD = Block Design, VP = Visual Puzzles, MR = Matrix Reasoning, FW = Figure Weights, DS = Digit Span, PS = Picture Span, CD = Coding, SS = Symbol Search. All standardized parameter estimates are significant at *p* < .001.

After metric invariance could be established, scalar invariance was tested by additionally constraining all subtest intercepts to be equal across the ADHD and the control group (M_4_). When compared to M_3_, however, a significant deterioration of the model fit of M_4_ was indicated by all fit indexes (ΔRMSEA = .044; ΔCFI = −.130; Δχ^2^ = 81.835, Δ*df* = 5, *p* < .001). As illustrated in Figure 1, not all subtest intercepts appeared to be similar across both groups; therefore, full scalar invariance was rejected. An additional partial scalar invariance model (M_4_^†^) was specified by analyzing and comparing all subtest intercepts across the ADHD and the control group. Since critical ratios for the pairwise parameter comparisons indicated that non-invariance could be attributed to unequal intercepts for the WISC-V subtests Vocabulary (VC), Digit Span (DS), Picture Span (PS), Coding (CD), and Symbol Search (SS), partial scalar invariance was tested by relaxing the according five subtest intercepts in M_4_. As soon as the subtest intercepts of VC, DS, PS, CD, and SS were allowed to vary across both groups, the fit indexes indicated no substantial decrease in the model fit of M_4_^†^ when compared to M_3_ (ΔRMSEA = .001; ΔCFI = −.002; Δχ^2^ = 5.405, Δ*df* = 2, *p* = .067).

**Figure 2:**
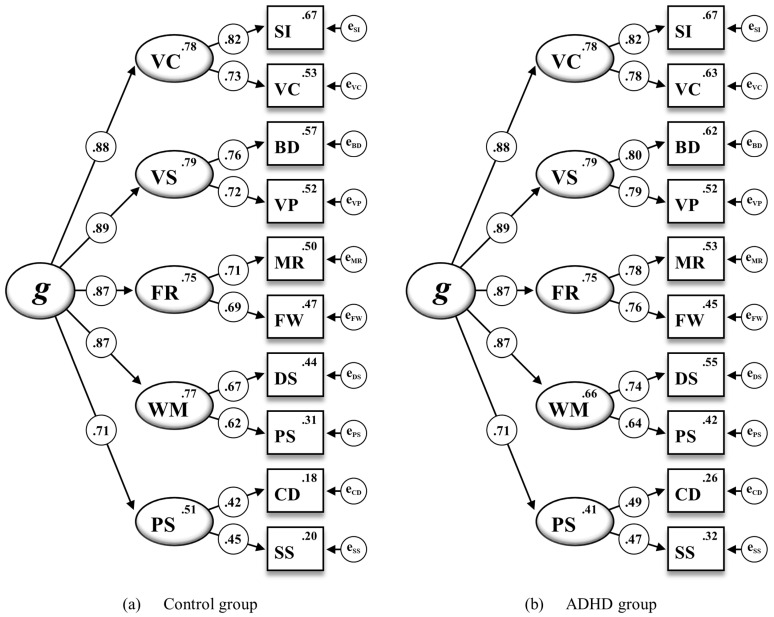
The WISC-V second-order five-factor model for the ADHD and control group including standardized estimations of all regression weights, subtest intercepts, and intercepts of the first-order factors for the 10 WISC-V primary subtests (M_5_^†^ in Table 3). Note. Second-order factor: *g* = General Intelligence. First-order factors: VC = Verbal Comprehension, VS = Visual Spatial, FR = Fluid Reasoning, WM = Working Memory, PS = Processing Speed. Subtest indicators: SI = Similarities, VC = Vocabulary, BD = Block Design, VP = Visual Puzzles, MR = Matrix Reasoning, FW = Figure Weights, DS = Digit Span, PS = Picture Span, CD = Coding, SS = Symbol Search. All standardized parameter estimates are significant at *p* < .001.

To complement the overall scalar invariance examination, a subsequent model was tested by additionally constraining all intercepts on the first-order factors to be equal across the ADHD and the control group (M_5_^†^). However, when compared to M_4_^†^, M_5_^†^ did result in a significantly worse model fit (ΔRMSEA = .027; ΔCFI = −.078; Δχ^2^ = 48.853, Δ*df* = 5, *p* < .001). As shown in Figure 2, some intercepts on the first-order factors turned out to vary across both groups, again suggesting that full scalar invariance could not be established. Following a critical ratio analysis indicating that non-invariance was likely due to nonequal intercepts of Working Memory (WM) and Processing Speed (PS), partial scalar invariance was again tested by relaxing the intercepts of both first-order factors in M_5_^††^. Once the intercepts of WM and PS were allowed to vary across both groups, fit indexes for M_5_^††^ did not indicate any substantial decrease in the model fit when compared to M_4_^†^ (ΔRMSEA = .001; ΔCFI = −.003; Δχ^2^ = 0.307, Δ*df* = 2, *p* = .858). Although full scalar invariance was rejected due to the non-invariant subtest intercepts of VC, DS, PS, CD, and SS (M_4_) as well as the non-invariant intercepts of the first-order factors WM and PS (M_5_^†^), scalar invariance could be partially established when allowing non-invariant parameters to vary across the ADHD and the control group. For the subsequent invariance model, the required parameter restrictions were thus based on the partial scalar invariance model (M_5_^††^).

In a final step, residual invariance was tested by constraining all error variances of the observed variables to be equal across both groups (M_6_^††^). As indicated by the fit indexes, the fit of M_6_^††^ to the data appeared to be acceptable and not significantly worse than the fit of M_5_^††^ (ΔRMSEA = .001; ΔCFI = −.003; Δχ^2^ = 13.493, Δ*df* = 10, *p* = .197). Therefore, residual invariance across both groups could be established.

### 3.3. Model Parameter Estimations and Model-Based Reliability

**Figure 3:**
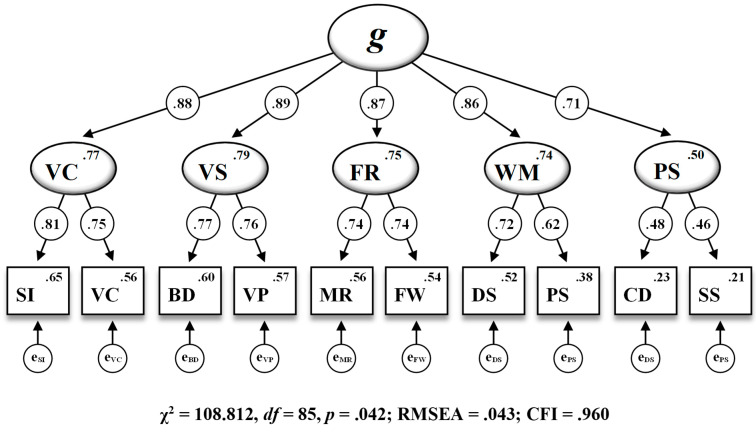
The WISC-V second-order five-factor model including standardized estimations of all regression weights, subtest intercepts, and intercepts of the first-order factors for the 10 WISC-V primary subtests (M_6_^††^ in Table 3). Note. Second-order factor: *g* = General Intelligence. First-order factors: VC = Verbal Comprehension, VS = Visual Spatial, FR = Fluid Reasoning, WM = Working Memory, PS = Processing Speed. Subtest indicators: SI = Similarities, VC = Vocabulary, BD = Block Design, VP = Visual Puzzles, MR = Matrix Reasoning, FW = Figure Weights, DS = Digit Span, PS = Picture Span, CD = Coding, SS = Symbol Search. RMSEA = root mean square error of approximation, CFI = comparative fit index. All standardized parameter estimates are significant at *p* < .001.

Standardized parameter estimates based on the residual invariance model (M_6_^††^) as the most restrictive model are displayed in Figure 3. Parameter estimations included in the present WISC-V second-order five-factor model for the most part appeared to be theoretically sound and consistent with the structural framework proposed by the test publishers. Factor loadings for subtest indicators on the corresponding first-order factors ranged from .46 for Symbol Search (SS) on Processing Speed (PS) to .81 for Similarities (SI) on Verbal Comprehension (VC). Among the associations between the first-order factors and the second-order factor, Visual Spatial (VS) featured the highest loading (.89) and Processing Speed (PS) had the lowest loading (.71) on General Intelligence (g).

Since the associations between subtest indicators and the second-order factor are fully mediated by the first-order factors due to the hierarchical nature of the WISC-V second-order five-factor model, the SL procedure was additionally conducted to derive direct associations between the second-order factor and the subtest indicators. This approach allowed for the evaluation of model-based reliability, construct replicability, and other sources of variance. As shown in Table 4, all ten subtest indicators featured acceptable loadings on both the second-order factor and their respective first-order factors according to an SL orthogonalized model framework. However, the proportion of common variance in the subtest indicators (ECV) that was uniquely explained by the first-order factors was rather small, ranging from .046 for PS to .058 for VC, compared to the ECV exclusively accounted for by the second-order factor (.741). Consequently, with approximately 74%, the greatest portion of explained common variance in the subtest indicators appeared to be specifically linked to the second-order factor. Values for the ω coefficient ranged from .362 for PS to .876 for *g*, suggesting that the common variances in the individual composite scores could likely be attributed to both the second-order factor (FSIQ) and most of the first-order factors. In line with the ECV estimates, ω_HS_ coefficients for all first-order-factors were found to be rather small, ranging from .155 for VS to .181 for PS, and thus falling below the threshold of .500 proposed by Reise et al. (2013). Since the ω_H_ coefficient value of .816 for the second-order factor was well above this criterion, the second-order factor appeared to be precisely measured by the underlying subtest indicators while being scarcely influenced by variability in other factors. Moreover, the *H* coefficient value of .860 for the second-order factor also indicated that this factor was well defined by the ten subtest indicators, whereas, by contrast, the minimum criterion of .700 (Hancock and Mueller 2001; Rodriguez et al. 2016) was not met by any of the first-order factors, with values ranging from .199 for PS to .245 for VC.

### 3.4. Group Comparisons

Table 5 shows the descriptive statistics for the ten WISC-V primary subtests, the five primary indexes, and the FSIQ across the ADHD and control group as well as t-test statistics and effect sizes for group comparisons.

The control group showed significantly higher mean subtests scores in the subtests VC, DS, CD, and SS as well as in the indexes VCI, WMI, PSI, and FSIQ. After adjusting α with the Bonferroni–Holm method, only WMI no longer differed significantly between the groups. The effects sizes for the subtests ranged from −0.56 for SS up to −0.76 for DS, which are, according to Cohen (1988), medium to large effect sizes. For the indexes, the effect sizes ranged from −0.45 for VCI up to −0.69 for PSI; therefore, the effect sizes were also medium to large.

## 4. Discussion

The aim of the present study was to investigate measurement invariance of the WISC-V across a group of children and adolescents with ADHD and a matched control group. For this purpose, confirmatory factor analyses (CFAs) were conducted on the hierarchical second-order five-factor model based on the ten primary subtests as proposed by Wechsler (Wechsler 2014a, 2017a). In a first step, the second-order five-factor model was examined using single-group CFAs for the ADHD and control group separately to assess the model’s overall fit in both groups. The WISC-V second-order five-factor model was found to sufficiently fit the data in both groups. Next, MGCFA was conducted on the same model structure in order to examine measurement invariance across both groups. Configural and metric invariance could be established, which are, according to Keith and Reynolds (2012), the most crucial invariance models of all. Since full scalar invariance had to be rejected, the intercepts of five subtest indicators (VC, DS, PS, CD, and SS), as well as the intercepts of two first-order factors (WM and PS) had to be relaxed to establish partial scalar invariance. In practice, it often appears to be difficult to achieve full invariance of subtest intercepts and intercepts of latent factors (Keith and Reynolds 2012); therefore, non-invariant intercepts are not infrequent (e.g., Immekus and Maller 2010). There is still an ongoing debate about the role of scalar invariance as a prerequisite for meaningful mean score comparisons across different populations or groups (Immekus and Maller 2010). According to Steinmetz (2013), unequal subtest intercepts can have a notable impact on disparities in factor means and the likelihood of significant differences. Some authors, such as Muthén and Asparouhov (2013), take a different approach and suggest that an evaluation of invariance should be based on approximate rather than full measurement invariance. By allowing for partial scalar invariance, it is at least possible to conclude that scores on specific latent variables are comparable across different groups while comparisons on others should be interpreted with caution.

Based on the suggestions of Byrne et al. (1989) that full scalar invariance is not necessarily a mandatory requirement for further tests of invariance, subsequent measurement invariance analyses were based on the partial scalar invariance model (M_5_^††^).

In due consideration of the complexity of the WISC-V model structure and the strictness of each measurement invariance test, it was concluded that the German WISC-V does at least feature full metric but only partial scalar invariance on the item and first-order factor level across the ADHD and the control group. Therefore, certain group comparisons can be seen as meaningful, as group differences in five out of ten WISC-V subtest scores are attributable to group differences in the underlying latent dimensions. Since two out of five first-order factors were found to be non-invariant across the ADHD and the control group, associations between the second-order factor General Intelligence (*g*) and the underlying first-order factors can be seen as different across both groups. Strictly speaking, scalar invariance for the first-order factors is a prerequisite for any group comparisons that are based on the mean scores of the associated second-order factor (Dimitrov 2010). However, an alternative and less strict interpretation of non-invariant first-order factors relates to the fact that mean scores of first-order factors are not observed scores and should thus not be treated in the same manner as scores of subtest indicators (Rudnev et al. 2018). Conclusively, a mean score of *g* should only be treated as a compensatory score representing a predefined combination of those first-order factors that were found to be invariant. It should also be noted that the FSIQ, as a representative measure for *g*, is computed based on subtest scores rather than index scores, which represent mean scores of the corresponding first-order factors. Since the hierarchical model framework (e.g., associations between subtest indicators and *g* are fully mediated by the first-order factors) differs from the actual WISC-V scoring framework (e.g., the FSIQ is directly derived from subtest scores); however, model-based reliability analysis on the measurement relations between the FSIQ and the subtest scores might be more indicative of the measurement properties of the FSIQ.

The standardized parameter estimates derived from the most restrictive residual invariance model were found to be theoretically robust and in accordance with the structural framework proposed by the test publishers (Wechsler 2014a, 2017a). Regarding the loadings of subtest indicators on the corresponding first-order factor, CD loaded the lowest on PS, and SI loaded the highest on VC. VS was the first-order factor that loaded the highest on the second-order factor *g*, whereas PS featured the lowest loading on *g*.

Moreover, the SL procedure was utilized to derive direct associations between the second-order factor and the subtest indicators. According to this SL orthogonalized model framework, all ten subtest indicators featured acceptable loadings on the second-order factor and the first-order factors. However, the proportion of common variance in the subtest indicators (ECV) that was uniquely explained by the first-order factors was rather small. Consequently, approximately 74% of the total explained shared variance among the subtest indicators was specifically associated with the second-order factor. This finding is consistent with previous studies on the factorial validity of the WISC-V, which also suggested that the second-order factor accounted for the greatest portion of common variance in the subtest indicators (Canivez et al. 2016, 2017; Fenollar-Cortés and Watkins 2019; Watkins et al. 2018). The ω_HS_ coefficients for all first-order factors were found to be rather small and fell below the threshold of .500 as proposed by Reise et al. (2013). In contrast, the ω_HS_ coefficient for the second-order factor was found to exceed this threshold. A sufficient reliability of *g* was also supported by the *H*-coefficient, which indicated that the second-order factor was well defined by the 10 subtest indicators; whereas, by contrast, the minimum criterion of .700 (Hancock and Mueller 2001; Rodriguez et al. 2016) was not met by any of the first-order factors. These results underpin the psychometric quality of the FSIQ, which is precisely measured by the underlying primary subtests. Since the WISC-V primary indexes might not be adequately defined by their corresponding subtest indicators, or may not seem to produce reliable index scores, clinical interpretations solely based on the primary indexes should only be made very cautiously.

As indicated by the group comparisons, the ADHD group performed significantly worse on the primary subtests VC, DS, CD, and SS. Significant group differences were also found for the VCI, PSI, and the FSIQ. The worse performance of children and adolescents with ADHD on WMI and PSI is in line with previous research highlighting a decreased working memory capacity in children with ADHD (Kasper et al. 2012; Martinussen et al. 2005) and limitations in processing speed (Gau and Huang 2014; Salum et al. 2014; Shanahan et al. 2006). The findings are partially consistent with studies showing the biggest group differences in performance on working memory tasks (e.g., Pauls et al. 2018) and in processing speeds (e.g., Yang et al. 2013) when comparing an ADHD group with a control group. Here, however, only performances on the subtest DS turned out to be lower in the ADHD group, which may be associated with deficits in the auditory working memory or phonological loop, while performances on PS did not differ between the groups.

The lower scores on the FSIQ found in children and adolescents with ADHD in the present sample were also consistent with findings from previous studies (Jiang et al. 2015; Pauls et al. 2018; Yang et al. 2013). It is thus assumed that deficits in working memory and processing speed may at least partly contribute to a lower overall IQ, but that lower performances on cognitive tests may be predominantly caused by ADHD symptoms such as impulsivity or inattentiveness. Consequently, deficits in working memory performance may be due to short-term memory problems in children with ADHD (Berlin et al. 2003; Sinha et al. 2008; Willcutt et al. 2005). In addition, children have also been found to perform more poorly than healthy controls on subtests measuring processing speed (Chhabildas et al. 2001; Shanahan et al. 2006) due to deficits in focused attention (Rucklidge and Tannock 2002), thus achieving lower scores on PSI (Calhoun and Mayes 2005).

This is also highlighted by the present measurement invariance analyses, which indicated rather non-invariant subtest intercepts on VC, DS, CD, and SS as well as on the first-order factors WM and PS. Quantitative group differences based on the corresponding subtest scores and index scores should only be interpreted with due caution, if at all. This is because failing to establish full scalar invariance in the present study may also be regarded as an indication for the subtest and index functioning varying across the ADHD and the control group. Therefore, low test scores of children and adolescents with ADHD on the corresponding WISC-V subtests and indexes might likely reflect symptomatic behavioral problems rather than true cognitive deficits, as the affected children and adolescents might perform below their cognitive capacity (Jepsen et al. 2009).

The findings of lower scores in the ADHD group on the subtest VC and the VCI are also in line with previous findings (Pauls et al. 2018). Language disorders are known to be more prevalent in children and adolescents with ADHD than in healthy ones (Sciberras et al. 2014). However, studies assessing verbal skills like listening comprehension, story retelling or semantic aspects of language, found that low performances on those verbal tasks of children with ADHD might be explained by general executive dysfunctions rather than an underlying deficit of linguistic functions in ADHD (McInnes et al. 2003; Purvis and Tannock 1997). Conclusively, we assume that the lower score of the ADHD group on the subtest VC is more likely due to common ADHD symptoms related to behavior, such as an impulsive response style or inattentiveness, than a general deficit in verbal ability. Pineda et al. (2007), for instance, assessed the verbal performance of ADHD and control children between 6 and 11 years of age using a comprehensive neuropsychological test battery and found that children with ADHD performed significantly worse in understanding verbal information, while listening was worse compared to healthy controls, especially on the verbal comprehension test measure.

### 4.1. Limitations

First, it should be noted that the total sample analyzed in the present study was rather small (*N* = 182) and could have led to less accurate parameter estimates in the structural equation models under investigation. Determining an adequate sample size for sufficient statistical power to provide generalizable structural equation models should be based on model-specific approaches, such as Monte Carlo data simulation techniques, rather than on rules-of-thumb (Wolf et al. 2013). Conclusively, model-specific sample size requirements might have resulted in a higher accuracy of parameter estimates and model fit statistics in both single- and multigroup confirmatory factor analyses of the present study. Therefore, future studies on measurement invariance should be based on large clinical sample sizes. It should also be noted that ADHD and ADD were not analyzed separately within the ADHD group. Moreover, about 69.2% of the ADHD group also had a comorbid learning disorder. Prevalence rates for comorbid learning disorders in the presence of ADHD are reported to range from 20 to 70% (Mattison and Mayes 2012; Mayes et al. 2000; Sexton et al. 2012; Yoshimasu et al. 2010). In a recent study, children with ADHD and a comorbid reading disorder and/or disorder of written expression showed even poorer performances on tasks of working memory than children with ADHD alone (Parke et al. 2020). Another study showed that children and adolescents with ADHD and a comorbid learning disorder performed worse on subtests associated with WMI and PSI and achieved lower scores on the FSIQ than children and adolescents with a learning disorder alone (Becker et al. 2021). It can thus be assumed that an ADHD-only sample might have shown fewer—or less pronounced—deficits in the WISC-V compared to the rather mixed ADHD sample in the present study.

Another issue relates to the hierarchical second-order five-factor model that was used as the baseline model in the present measurement invariance analyses. The WISC-V factor structure is still part of an ongoing debate (Dombrowski et al. 2022a) and numerous studies have already examined the factorial validity of the WISC-V, including not only the American (Canivez et al. 2017) but also the British (Canivez et al. 2019), Canadian (Watkins et al. 2018), French (Lecerf and Canivez 2018), Spanish (Fenollar-Cortés and Watkins 2019), and German (Canivez et al. 2021; Pauls and Daseking 2021) versions. However, there is still no agreement on whether the five-factor model or a four-factor model on the one hand, or whether a hierarchical or a bifactor model on the other hand represents the WISC-V best (see for example Canivez et al. 2017; Dombrowski et al. 2018). Canivez et al. (2020) examined the factor structure of the WISC-V in a heterogeneous clinical sample including a large proportion of children and adolescents with an ADHD diagnosis (Canivez et al. 2020). Exploratory factor analysis (EFA) indicated that a four-factor model fitted the empirical data best, and that confirmatory factor analysis (CFA) supported a bifactor model with four group factors. Further studies have also examined the factor structure of the WISC-IV when administered to children and adolescents with ADHD (Fenollar-Cortés et al. 2019; Gomez et al. 2016; Styck and Watkins 2017; Thaler et al. 2015; Yang et al. 2013). The results of these studies have supported the four-factor model structure; however, there is also evidence for a five-factor model (Thaler et al. 2015) characterized by either a higher-order (Styck and Watkins 2017) or a bifactorial structure (Gomez et al. 2016). Thus, an extensive investigation of alternative model solutions (e.g., bifactor models) should be performed in future studies on ADHD samples.

The present analyses of model-based reliability supported the previously suggested dominance of *g* and the limited unique measurement of the group factors of the WISC-V (Canivez et al. 2020). In particular, model-based reliability and construct replicability coefficients for *g* turned out to be satisfactory, thus justifying a meaningful interpretation of an overall measure such as the FSIQ. However, this is only true if the calculation of the FSIQ is based on all ten WISC-V primary subtests and not on seven out of ten primary subtest scores, as described in all versions of the WISC-V. Thus, interpretability of *g* cannot be equally guaranteed for the FSIQ, as this measure might under- or over-estimate true levels of General Intelligence. Regarding the WISC-V primary indexes, reliability and replicability coefficients for the group factors appeared to be too low to warrant reliable measures for specific cognitive dimensions. Therefore, researchers and clinicians should be cautious when interpreting the WISC-V primary index scores individually. Diagnostic decision-making should be predominantly based upon the FSIQ.

### 4.2. Conclusions and Implications for Practice

The factor structure of the WISC-V proposed by the test publishers (Wechsler 2014a, 2017a) was found to sufficiently fit the data of both the ADHD and the control group in the present study. Five out of ten WISC-V primary subtests were observed to be fully invariant across these groups. However, since five subtest intercepts appeared to be non-invariant, the corresponding index scores for VCI, WMI, and PSI cannot be suggested to be comparable across children and adolescents with ADHD and healthy ones. This may then diminish the usefulness of the associated primary subtests VC (VCI), DS (WMI) as well as CD and SS (PSI) in measuring the underlying latent abilities. Since children and adolescents with ADHD not only achieved significantly lower scores on VC, DS, CD, and SS than healthy controls, and intercepts for two of the corresponding first-order factors were also found to be non-invariant across both groups, these primary subtests appeared to be harder for individuals with ADHD than would be expected for the corresponding scores on the underlying latent factor. It could thus be assumed that such group differences can be a result of the critical subtests measuring slightly different cognitive subdimensions for the compared groups.

As described previously, the behavioral problems and cognitive deficits of children and adolescents with ADHD may likely manifest themselves in performances primarily associated with working memory and processing speed (Gau and Huang 2014; Kasper et al. 2012; Martinussen et al. 2005; Salum et al. 2014; Shanahan et al. 2006). Even though the according WISC-V primary indexes WMI und PSI should only be interpreted with caution and a reliable profile analysis is not fully warranted (as has already been shown by previous research), an analysis of the underlying cognitive profiles may be used as an orientation for identifying individual strengths and weaknesses of those affected. Although differential diagnostic decision-making cannot, and should not, be based upon those cognitive profiles (Dombrowski et al. 2022b; McGill et al. 2018), profile analyses may provide additional anamnestic information about the cause of possible cognitive deficits, depending on the diagnostic question. Assuming that symptomatic behavioral problems in ADHD may substantially affect performances on the WISC-V subtests, future studies should clarify whether measurement invariance of the WISC-V can still be established when investigating different ADHD subpopulations with varying levels of symptom severity (e.g., after partial symptom remission).

Since the present study could, at least to some extent, demonstrate sufficient levels of measurement invariance of the German WISC-V across the ADHD and control group, it may be considered a partially suitable test battery for measuring specific intellectual abilities in children and adolescents with ADHD. Yet, there are some substantial limitations regarding the interpretation of single non-invariant subtest and index scores that need to be considered cautiously in future research and clinical practice. Most importantly, clinical interpretations based on the WISC-V primary indexes and the according cognitive profiles are only admissible to a limited extent. When interpreting WISC-V test scores, especially those derived from non-invariant subtests and indexes, practitioners should always keep in mind that children and adolescents with ADHD are likely to perform below their cognitive capacity in the WISC-V and may thus fall short of their potential due to their specific symptomatology.

## Figures and Tables

**Table 1:** Demographic description of the ADHD group and control group.

	ADHD Group (*n* = 91)	Control Group (*n* = 91)
Age *M*(*SD*)	10.83 (2.46)	10.48 (2.45)
	*n* (%)	*n* (%)
Sex (female)	26 (28.6)	26 (28.6)
Migration background	27 (29.7)	26 (28.6)
Type of school (*n* and %)		
Primary school	47 (52.2)	47 (52.2)
Secondary school, graduation after 9th grade (German: Hauptschule)	3 (3.3)	5 (5.5)
Secondary school, graduation after 10th grade (German: Realschule)	0 (0)	6 (6.6)
Grammar school, graduation after 12th or 13th grade, university entrance degree (German: Gymnasium)	12 (13.2)	16 (17.6)
Comprehensive school, different kinds of degrees can be obtained after 9th/10th or 12th/13th grade (German: Gesamtschule)	25 (27.5)	16 (17.6)
Special school (German: Förderschule)	4 (4.4)	1 (1.1)
Parental education (*n* and %)		
Lower education level	10 (11.0)	8 (8.8)
Medium education level	31 (34.1)	33 (36.3)
High education level	23 (25.3)	22 (24.2)
Highest education level	27 (29.7)	28 (30.7)

Note. Parental education is defined as the highest level of education achieved by either one parent or both (low educational level = no diploma or school certificate after 9th grade, medium educational level = school certificate after 10th grade, high educational level = university entrance qualification/certificate after 12th or 13th grade, and highest educational level = college/university degree).

**Table 2:** Goodness-of-fit indexes of the single-group confirmatory factor analyses on the WISC-V second-order five-factor model (Phase 1).

Group		Indexes of Model Fit								
		χ²	*df*	χ²/*df*	*p*	SRMR	RMSEA	(90% CI)	CFI	AIC
ADHD	(*n* = 91)	41.423	30	1.381	.080	.050	.065	.011–.110	.961	91.423
Control	(*n* = 91)	48.265	30	1.609	.019	.045	.072	.024–.114	.958	98.265

Note. The WISC-V second-order five-factor model includes ten subtest indicators (SI, VC, BD, VP, MR, FW, DS, PS, CD, and SS), five first-order factors (VC, VS, FR, WM, and PS), and one second-order factor (*g*). SRMR = Standardized root mean square residual, RMSEA = Root mean square error of approximation, (90% CI) = Confidence interval for RMSEA, CFI = Comparative fit index, AIC = Akaike information criterion.

**Table 3:** Multigroup goodness-of-fit indexes and invariance model comparisons for the WISC-V second-order five-factor model (Phase 2).

Invariance Model	Indexes of Model Fit								Model Comparison					
	χ²	*df*	χ²/*df*	SRMR	RMSEA	(90% CI)	CFI	AIC	Comparison	ΔRMSEA	ΔCFI	Δχ²	Δ*df*	*p*
M_1_: Configural	79.535	62	1.283	.055	.038	.000–.061	.971	215.535						
M_2_: Metric (1st order)	84.244	67	1.257	.060	.039	.000–.062	.970	210.244	M_2_ vs. M_1_	.001	−.001	4.709	5	.452
M_3_: Metric (2nd order)	89.607	71	1.262	.065	.040	.000–.063	.968	207.607	M_3_ vs. M_2_	.001	−.002	5.363	4	.252
M_4_: Scalar (obs.)	171.437	76	2.256	.189	.084	.067–.100	.838	279.437	M_4_ vs. M_3_	.044	−.130	81.835	5	<.001
M_4_^†^: Partial scalar (obs.)	95.012	73	1.302	.064	.041	.008–.062	.966	209.012	M_4_^†^ vs. M_3_	.001	−.002	5.405	2	.067
M_5_^†^: Scalar (lat.)	143.866	78	1.844	.083	.068	.051–.086	.888	247.866	M_5_^†^ vs. M_4_^†^	.027	−.078	48.853	5	<.001
M_5_^††^: Partial Scalar (lat.)	95.319	75	1.271	.064	.042	.000–.061	.963	205.319	M_5_^††^ vs. M_4_^†^	.001	−.003	0.307	2	.858
M_6_^††^: Residual	108.812	85	1.280	.065	.043	.008–.060	.960	198.812	M_6_^††^ vs. M_5_^††^	.001	−.003	13.493	10	.197

Note. M_1_ = unconstrained baseline model, M_2_ = model with equal loadings on all first-order factors, M_3_ = M_2_ with equal loadings on the second-order factor, M_4_ = M_3_ with equal subtest intercepts, M_4_^†^ = M_4_ with five relaxed subtest intercepts (VC, DS, PS, CD, and SS), M_5_^†^ = M_4_^†^ with equal intercepts of first-order factors, M_5_^††^ = M_5_^†^ with two relaxed intercepts of first-order factors (WM and PS), M_6_^††^ = M_5_^††^ with equal error variances on all subtests. SRMR = standardized root mean square residual, RMSEA = root mean square error of approximation, (90% CI) = confidence interval for RMSEA, CFI = comparative fit index, AIC = Akaike information criterion, ΔRMSEA = difference in RMSEA between compared models, ΔCFI = difference in CFI between compared models, Δχ^2^ = chi-square difference between compared models, Δ*df* = difference in degrees of freedom between compared models.

**Table 4:** Sources of variance in the 10 WISC-V primary subtests for the control and ADHD samples according to the SL orthogonalized WISC-V second-order five-factor model (M_6_^††^).

WISC-V Subtest	*g*		VC		VS		FR		WM		PS			
	*b*	*S* ^2^	*b*	*S* ^2^	*b*	*S* ^2^	*b*	*S* ^2^	*b*	*S* ^2^	*b*	*S* ^2^	*h* ^2^	*u* ^2^
SI	.709	.505	.388	.151									.653	.347
VC	.654	.428	.358	.128									.556	.444
BD	.688	.473			.354	.125							.599	.401
VP	.672	.452			.346	.120							.571	.429
MR	.645	.416					.369	.136					.552	.448
FW	.639	.408					.365	.133					.542	.458
DS	.622	.387							.369	.136			.523	.477
PS	.533	.284							.316	.100			.384	.616
CD	.339	.115									.339	.115	.230	.770
SS	.326	.106									.326	.106	.213	.787
Total *S*^2^		.357		.028		.025		.027		.024		.022	.482	.518
ECV		.741		.058		.051		.056		.049		.046		
ω		.876		.753		.738		.707		.623		.362		
ω_H_/ω_HS_		.816		.174		.155		.174		.162		.181		
Relative ω		.932		.231		.209		.246		.260		.500		
*H*		.860		.245		.218		.237		.212		.199		
PUC		.889												

Note. General factor: *g* = General Intelligence. Group factors: VC = Verbal Comprehension, VS = Visual Spatial, FR = Fluid Reasoning, WM = Working Memory, PS = Processing Speed. Subtest indicators: SI = Similarities, VC = Vocabulary, BD = Block Design, VP = Visual Puzzles, MR = Matrix Reasoning, FW = Figure Weights, DS = Digit Span, PS = Picture Span, CD = Coding, SS = Symbol Search. *b* = factor loading (significant at *p* < .05), *S*^2^ = explained variance, *h*^2^ = communality, *u*^2^ = unique variance, ECV = explained common variance, ω = omega coefficient, ω_H_ = omega-hierarchical coefficient (general factor), ω_HS_ = omega-hierarchical coefficient (group factors), H = replicability index (construct reliability), PUC = percentage of uncontaminated correlations.

**Table 5:** Mean, standard deviation and t-test statistics for group comparisons on the ten WISC-V primary subtests and indexes.

WISC-V Subtest /Index	ADHD Group *n* = 91		Control Group *n* = 91		Test Statistics				
	*M*	*SD*	*M*	*SD*	*t*	*(df)*	*p*	*p_cor._*	*d*
SI	10.27	2.63	10.87	2.68	1.51	(180)	.133	>.999	−0.23
VC	9.58	2.75	11.15	2.77	3.84	(180)	<.001	.016	−0.57
BD	9.78	2.62	10.36	2.99	1.39	(180)	.164	>.999	−0.21
VP	10.56	2.71	10.35	2.82	−0.51	(180)	.611	>.999	0.07
MR	9.71	2.58	10.10	2.84	0.96	(180)	.339	>.999	−0.14
FW	10.52	2.58	10.47	2.91	−0.11	(180)	.914	>.999	0.02
DS	8.44	2.67	10.62	3.04	5.38	(170) ^a^	<.001	.016	−0.76
PS	10.64	2.78	10.10	3.11	−1.23	(180)	.220	>.999	0.18
CD	8.60	2.53	10.58	3.15	4.66	(180)	<.001	.016	−0.69
SS	8.71	2.73	10.25	2.82	3.74	(180)	<.001	.016	−0.56
VCI	99.48	13.48	105.47	13.18	3.03	(180)	.003	.030	−0.45
VSI	100.73	13.47	101.91	14.34	0.58	(180)	.566	>.999	−0.09
FRI	100.70	13.24	101.71	13.90	0.50	(180)	.616	>.999	−0.07
WMI	96.99	12.75	102.70	15.07	2.45	(180)	.015	.135	−0.41
PSI	92.63	12.64	102.45	15.44	4.69	(180)	<.001	.016	−0.69
FSIQ	96.81	12.34	104.21	13.78	3.82	(180)	<.001	.016	−0.57

Note. ^a^ An adjustment to the degrees of freedom using the Welch–Satterthwaite method was made due to unequal group variances as shown by the Levene test. Subtest indicators: SI = Similarities, VC = Vocabulary, BD = Block Design, VP = Visual Puzzles, MR = Matrix Reasoning, FW = Figure Weights, DS = Digit Span, PS = Picture Span, CD = Coding, SS = Symbol Search. Primary indexes indicators: VCI = Verbal Comprehension Index, VSI = Visual Spatial Index, FRI = Fluid Reasoning Index, WMI = Working Memory Index, PSI = Processing Speed Index, FSIQ = Full Scale IQ. *p_cor_*_._ = *p* corrected based on the Bonferroni–Holm method. *d* = Cohen’s d.

## Data Availability

Since this study was carried out as part of the general health service research (Versorgungsforschung), the data are not publicly available.
